# Drivers of habitat quality for a reintroduced elk herd

**DOI:** 10.1038/s41598-022-25058-9

**Published:** 2022-12-05

**Authors:** Braiden A. Quinlan, Jacalyn P. Rosenberger, David M. Kalb, Heather N. Abernathy, Emily D. Thorne, W. Mark Ford, Michael J. Cherry

**Affiliations:** 1 Department of Fish and Wildlife Conservation, Virginia Polytechnic Institute and State University, Blacksburg, VA 24061 USA; 2 Virginia Department of Wildlife Resources, Marion, VA 24354 USA; 3 Rhode Island Department of Environmental Management, Providence, RI 02908 USA; 4 Caesar Kleberg Wildlife Research Institute, Texas A&M University - Kingsville, Kingsville, TX 78363 USA; 5 U.S. Geological Survey, Virginia Cooperative Fish and Wildlife Research Unit, Blacksburg, VA 24061 USA

**Keywords:** Ecology, Conservation biology, Forest ecology, Grassland ecology, Restoration ecology

## Abstract

Understanding spatiotemporal variation in habitat quality is essential for guiding wildlife reintroduction and restoration programs. The habitat productivity hypothesis posits that home range size is inversely related to habitat quality. Thus, home range size may be used as a proxy for habitat quality and can identify important land cover features for a recovering species. We sought to quantify variation in home range size across the biological cycle (seasons) for a reintroduced elk (*Cervus canadensis*) population in southwestern Virginia, USA and quantify habitat quality by linking home range sizes to the land cover types they contain using linear mixed-effects models. We found mean home range size was largest during late gestation for female elk. Additionally, throughout the year, smaller home ranges were associated with larger proportions of non-forested habitats whereas forested habitats were generally the opposite. However, both presumed poor- and high-quality habitats influenced female elk space use. Our approach revealed spatial variation in habitat quality for a recovering elk herd, demonstrated the importance of non-forested habitats to elk, can guide decisions regarding the location of future elk reintroduction programs, and serve as a model for evaluating habitat quality associated with wildlife reintroductions.

## Introduction

Understanding spatiotemporal variation in resource quality is a cornerstone of animal ecology because such variation influences animal distribution, behavior, and fitness^1^. To minimize energetic requirements, an animal will often occupy the minimum amount of space that it needs to carry out its life history functions^2^. Considering the habitat productivity hypothesis—that posits resource quality, aggregating all factors of habitat quality, is negatively associated with space use^2,3^—higher quality or clumped resources often result in less space use due to more efficient movements associated with resources (e.g., searching for food^4,5^, mates^6,7^, and cover^8^).

Across species, home range size varies amongst populations and individuals. However, spatiotemporal comparisons of space use between or within populations can be used as a proxy for seasonal resource availability^9^. There are numerous drivers of space use which dictate resource use and quality: foraging^10,11^, resting^12^, reproductive cycles^13,14^, predator avoidance^10,13^, inter- and intraspecific resource competition^15^, thermoregulation^12,16–18^, and anthropogenic disturbance^10,19–21^. These drivers of space use can influence home range size solely or in an additive manner contingent upon individual characteristics, such as an animal’s sex, age, body size, weather, region, and population^22^. Linking the composition of home ranges to their sizes can reveal higher and lower quality species-specific resources regardless of the driver^3,23^, and therefore allows for inference between temporal variation in resource availability and use, and how animals track resources through space and time.

Reintroductions are an important wildlife management tool and have reversed declines for numerous species^24^. Yet, reintroductions often require empirical knowledge regarding the niche and movements of reintroduced species within the restoration zone. For example, identifying suitable reintroduction sites depends on the understanding of reintroduction landscape and habitat quality for the focal species^25^. Continued habitat quality assessments for reintroduced species may alleviate gaps in knowledge and aid in the longevity of the population^26^. However, when reintroductions have the potential to generate human-wildlife conflict (e.g., crop damage by herbivores)^27,28^, understanding space use and range expansion beyond the reintroduction zone becomes vital to minimize and mitigate conflict^29,30^. Quantifying behavioral responses of reintroduced species to spatiotemporal variability in resource quality may assist in mitigating conflict and ensuring population persistence as such empirical knowledge allows predictions regarding range expansion beyond the reintroduction site^31^.

Elk (*Cervus canadensis*) were once distributed across North America prior to their extirpation east of the 90th meridian west by the late 19th century^32^. Following European settlement, elk populations declined from overharvest and habitat loss^33,34^. At the turn of the 20th Century, elk from western populations were reintroduced to the former range of elk in the eastern United States of America (USA)^34^. However, elk populations in western North America use open habitats^19^, and following numerous failed reintroductions, a few successful reintroductions highlighted that elk in the eastern USA require non-forest habitats embedded in the forest matrix^10,11,35–37^. In the central Appalachian Mountain coalfield regions (hereafter Coalfields), these sites largely are reclaimed coal surface mines (hereafter reclaimed mines) functioning as large (> 200 ha) contiguous grassland patches^34^. Reclaimed mines provide grassy forage quickly following the reclamation process^38^. Because of this, in both the Kentucky and Tennessee Coalfields, elk utilize these sites^11,35,36^. Due to numerous and very large reclaimed mines, elk populations in eastern Kentucky increased beyond initial expectation^39^ and as the population grew, individuals immigrated to surrounding states including Virginia. This occurrence spurred the Virginia Department of Wildlife Resources (VDWR) to create an Elk Management Zone (VEMZ) encompassing Buchanan, Dickenson, and Wise counties in the far southwestern portion of the state where elk harvest was illegal^33^. To accelerate the establishment of elk in the state, from 2012 to 2014, VDWR reintroduced 75 elk from Kentucky into Buchanan County^33^.

The abundance of forest habitats in the eastern reintroduction sites may influence how elk respond to thermal stress, predation risk or human activity, and forage availability thereby affecting home range size. With increasing ambient temperatures, elk often require some forest to provide shade to remain below their upper critical temperature^17^. Cook et al.^40^ found that elk lacking dense forest cover during the summer in Oregon in the western USA required greater water intake, an issue of concern as well in warmer southeastern North American climates. In colder winter climates, elk can use forest cover, particularly conifers, as means to retain radiant heat^12^. During colder weather at temperatures below an elk’s thermal neutral zone, particularly when cover is not present, elk expend more energy regulating their temperature, i.e., through shivering^12,41^. However, in many of the elk reintroduction sites in the Coalfields, winters are far milder than experienced in much of western North America, and elk may not require forests for thermal retention. Still, human activity and predation risk can force elk to seek cover in forested habitats. Part of the western North American population of elk, female elk in Montana increased their use of forested habitat prior to and during a hunting period^19^, and elk will also move to forested areas to avoid predation^42,43^. During spring and summer, elk may use forests and edge habitats as cover for calving sites^14^ with highly available woody browse^14,36,44^. Additionally, in the southern Appalachian and presumably the central Appalachian Mountains in the eastern USA, during the fall, elk will use forests to consume hard mast (i.e., acorns)^35^. In the Coalfields, elk simply may use forests more because of the abundant, highly palatable woody vegetation foliage therein^45^.

With the reintroduction of western elk more adapted for open habitats into the forested eastern USA, our objective was to understand the drivers of habitat quality in the eastern system. Eastern elk may be forced to travel greater distances to satisfy their critical needs, or they may be constrained by the variation in availability of resources. Thus, considering the habitat productivity hypothesis, we hypothesized that elk home ranges would vary as a function of habitat composition and spatiotemporal variation in resources across the annual biological cycle. Given vegetation composition at the reintroduction zone, we predicted (1) elk home range size would be inversely related to the proportion of non-forested habitats and positively related to forested habitats because they are grazers and (2) elk home range size would increase during the dormant season (i.e., winter) in search for sufficient forage to meet energetic needs.

## Methods

### Ethical statements for methods

(i) We confirm all experimental protocols were approved by the Virginia Department of Wildlife Resources.

(ii) We confirm all elk were captured by the Virginia Department of Wildlife Resources under their agency institutional animal care and use committee and in accordance with the American Society of Mammalogists guidelines^46^.

(iii) We confirm all methods are reported in accordance with the ARRIVE 10 Essential Guidelines where applicable. ARRIVE Essential Guidelines 1, 2, 3, 4, 5, 6, 8, and 9 are not directly applicable to our research as they reference experimental study designs, and our work was observational in design^47^.

### Study area

**Figure 1:**
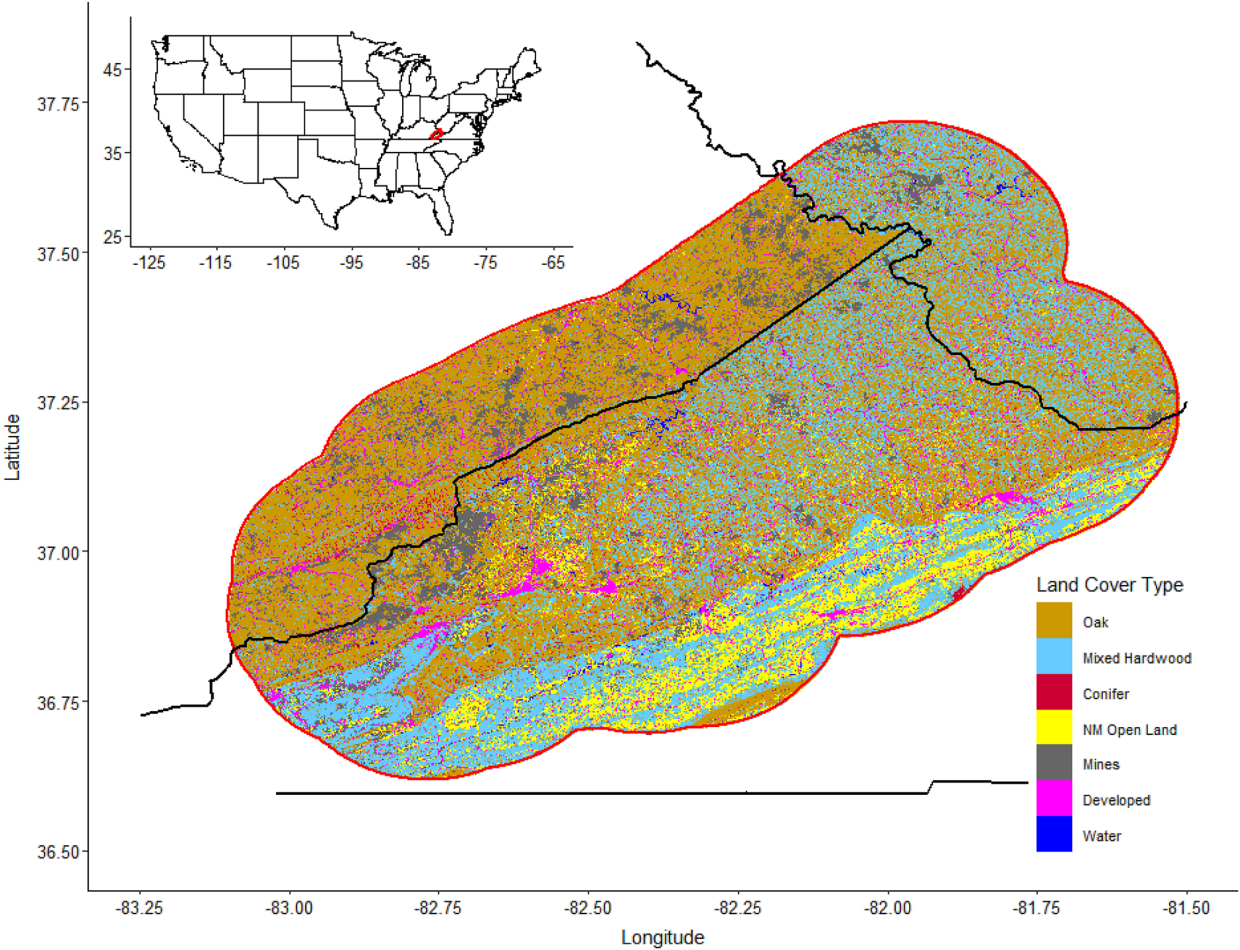
Our study area comprised of a 20 km buffer around the Virginia Elk Management Zone (Buchanan, Dickenson, and Wise counties, Virginia, USA) including surrounding portions of eastern Kentucky and southern West Virginia in the central Appalachian Mountains in the eastern USA, 2012–2019. Land cover types included: oak, mixed hardwood, conifer, non-mined (NM) open land, surface mines, developed, and water.

We used a 20-km (km) buffer around the VEMZ to establish a study area; encompassing 10,326.4 km^2^ of southwestern Virginia and small portions of eastern Kentucky and southern West Virginia (Fig. 1). The VEMZ largely occurs in the Cumberland Mountains portion of the Appalachian Plateau physiographic sub-province of the central Appalachian Mountains^48^. This region is dominated by second- and third-growth Appalachian oak (*Quercus* spp.) and diverse cove and mixed mesophytic hardwood forest types that include admixtures of eastern hemlock (*Tsuga canadensis*)*,* American beech (*Fagus grandifolia*)*,* yellow-poplar (*Liriodendron tulipifera*)*,* pignut hickory (*Carya glabra*)*,* white ash (*Fraxinus americana*)*,* black cherry (*Prunus serotina*), basswood (*Tilia americana*), northern red oak (*Quercus rubra*), and maples (*Acer* spp.)^49,50^. The central Appalachian Mountains has a long history of both deep and surface coal mining, with the latter being an increasingly dominant land over the past few decades (approximately 7% of total land cover), second to forest cover (approximately 75%) in extent^51,52^. In our study area, surface mine size varies greatly (< 200 to > 5000 ha). Elevation in our study area ranges from 185 to 1436 m above sea level and topography is characterized by steep slopes with narrow incised valleys. Thirty-year average monthly temperatures ranged from 0.9 °C in January to 22.7 °C in July^53^. Thirty-year average monthly precipitation ranged from 6.6 cm in November to 14.5 cm in July, with greatest snowfall occurring during January averaging 15.7 cm per year^53^.

We deployed global positioning system (GPS) collars (ATS G5-2D Iridium) on elk upon release to the VMEZ (2012: n = 9; 2013: n = 2; 2014: n = 23)^33^, or subsequently thereafter from opportunistic captures (2019: n = 16; 2020: n = 7, 2021: n = 2). Three previously collared females were re-collared during 2019 and 2020. We accumulated GPS data for 34 female elk from June 2012 to February 2017 and 25 female elk from March 2019 to February 2022. We then separated locations into four seasonal distinctions: mid-gestation (12/01–02/28), late gestation (03/01–05/31), calving (06/01–08/31), and harems (09/01–11/30) largely following the winter, spring, summer, and fall seasons. We chose these distinctions based on their biological and energetic significance for elk while also dividing the year into as equal segments as possible. Specifically, mid-gestation is characterized by the onset of mature bulls forming bachelor groups, and cows are partially through their gestational period (range: two to five months) and ending prior to spring green-up. Late gestation is characterized as at or before the first signs of green-up and ending with the start of the parturition (birthing) period. The calving season began during parturition, continuing through the summer, and ending when bulls rejoin matriarchal groups. Finally, the harems season is characterized by bulls forming harems through the breeding season (rut, early October) and ending just prior to bulls leaving their harems.

We used land cover from National Gap Analysis Project Land Cover Data 2011 (GAP) version 3.0^54^ and the Northeastern Terrestrial Habitats (NTH) layer^55^, both at 30 m resolution, to quantify land cover types across our study area. We reclassified land in ArcMap 10.8.1^56^ into seven distinct types based on the descriptions of the finer-scale land cover types following Ford et al.^57^ and Kniowski and Ford^58^. Reclassified land cover types included: oak forests, cove and mixed mesophytic hardwoods, conifer (*Pinus* spp.), mines (quarries, mines, gravel pits, oil wells, disturbed and barren lands associated with mining), non-mining (NM) open land (pasture, cultivated, and other non-mine open lands), developed (communities, roads, and infrastructure), and water (lakes, reservoirs, large rivers, and associated riparian area). We derived our mines land cover for the entire study area and all of Kentucky’s land cover from GAP and the remaining habitat types for the rest of the study area from NTH. We combined these land cover raster layers and used the resulting raster as the land cover data for this analysis (Fig. 1).

Prior to analysis, we removed duplicate locations and time errors from our GPS data. We parsed GPS locations by season and filtered fix-rates to a maximum of 24-h. We retained animals that collected GPS locations for > 75% of the respective season, and at least 60 locations within that season. Moreover, we removed outlier GPS locations that fell outside of the upper 97% of distance between consecutive locations. To quantify elk space use, we created continuous-time movement models for each individual, each season, each year using *ctmm.guess* to generate movement model initiation points then *ctmm.select* to fit parsimonious movement models using the ctmm package^59^ in program R version 4.0.5^60^. Using individual-level continuous-time movement models, we generated 95% occurrence distributions for each elk using the *occurrence* function^59^. Here we refer to ‘occurrence’ as an observed moment in time (i.e., GPS location) and ‘occurrence distribution’ (hereafter home range) as the area that results in the uncertainty of a realized movement path during the observation window (i.e., season-year)^61^. We used occurrence distributions over other home range estimators because we wanted to delineate what habitat types elk were utilizing within tight-fitting seasonal home ranges for strongest composition and size comparisons across seasons without extrapolating beyond seasonal boundaries^61^. To determine if elk home range sizes varied across seasons, we first checked for normality of our data by conducting a Shapiro–Wilk test of normality (function *shapiro.test*)^60^, and when it failed, we ran a Kruskal–Wallis test (function *kruskal.test*)^60^ as a non-parametric alternative to one-way ANOVA. We used a pairwise Wilcoxon rank sum test (function *pairwise.wilcox.test*)^60^ using the output from the Kruskal–Wallis test to compare averaged seasonal home range sizes across all individuals.

**Figure 2:**
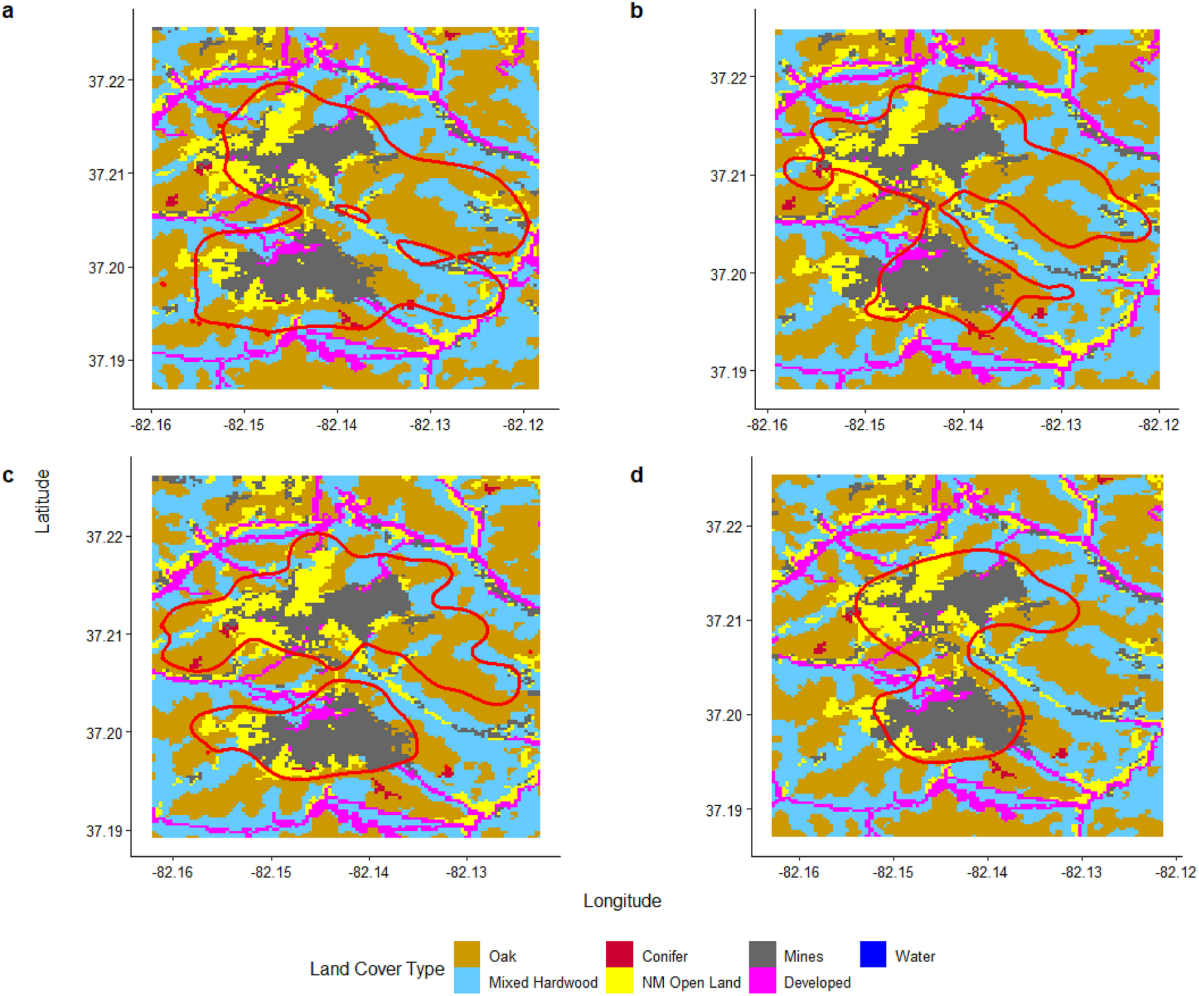
Home ranges (red lines) overlaid on land cover classifications for elk B453 during late gestation (**a**), calving (**b**), harems (**c**), and mid-gestation (**d**) seasons, 2015 in southwestern Virginia, USA. Notably, this elk had her largest home range during late gestation, followed by harems, then calving, and lastly mid-gestation. During late gestation, her home range was 34% oak, 24% mixed hardwood, 14% non-mined (NM) open land, and 24% mines for the main four land cover types. These percentages shifted slightly during calving to 32% oak, 17% mixed hardwood, 15% NM open, and 32% mines. During harems season, B453 had similar proportions to calving with 33% oak, 15% mixed hardwood, 18% NM open, and 30% mines. However, the largest change in proportions came during mid-gestation season where B453 centered her home range on mines (43%) and NM open land (20%) with much lower percentages of oak (22%) and mixed hardwood (11%).

To calculate land cover proportions within home ranges, we extracted values from the land cover raster within each individual’s home range using the *raster* package in program R (Fig. 2)^62^. We summed the cells for each land cover type and divided each by the total number of cells of all land cover types within the home range polygon to calculate a proportion of land cover types within each animal’s home range (Fig. 2). We scaled and centered the proportions of land cover types with means of zero and standard deviations of one^63^. We tested for multicollinearity across all land cover proportions and values that were |r|> 0.5 were not included in the same model as those values were considered moderate to high in collinearity.

To quantify elk home range size as a function of the four main land cover types in our study area (oak forests, mixed hardwoods, NM open land, and mines), we used linear mixed-effects models using the *lmer* function from the lmerTest package^64^ in program R. We considered land cover types as fixed effects and individual elk as random effects as some individuals were monitored multiple years. We conducted two analyses corresponding to two biological scales—seasonal and yearly comparisons. For the year-round comparisons, we incorporated all individual’s seasonal home ranges and associated proportions of land cover types in each model. For seasonal comparisons, we used the seasonal home ranges and their associated proportions of land cover types. In both analyses, we used an Akaike information criterion (AIC) information-theoretic approach selecting the model with the lowest delta AIC as the top model^14,65^. We identified informative parameters using p-values provided by the summary of the *lmer* function from the lmerTest package in program R which uses Satterthwaite’s approximation for degrees of freedom to identify informative parameters at alpha = 0.05^64^.

## Results

Overall, we found that the VEMZ was 75% forested, dominated by oak forest (45.8%), followed by cove and mixed mesophytic hardwoods (27.0%). NM open lands comprised 9.7% and surface mines accounted for 9.1%. The other land cover types were conifer forests (1.3%), developed (6.7%), and water (0.5%).

### Home range sizes

From 104,174 GPS locations of female elk in the VEMZ during our survey period, we had 355 home ranges varying by season: late gestation (79), calving (105), harems (91), and mid-gestation (80). The largest observed seasonal home ranges of female elk occurred during late gestation (875.9 ± SE 79.4 ha) whereas the smallest were mid-gestation (586.4 ± SE 47.3 ha) (Table 1). Mean home range size was 627.9 ± SE 53.5 ha during the calving period and 694.1 ± SE 45.2 ha during the harems period (Table 1). The pairwise Wilcoxon rank sum test showed no significant differences (p > 0.05) in home range sizes among calving, harems, and mid-gestation seasons. However, home range sizes during late gestation were significantly larger (p < 0.05) than calving and mid-gestation seasons.

**Table 1:** Average biological season home range sizes with standard errors (SE) and 95% confidence limits (CL) for female elk in southwestern Virginia, USA from June 2012–February 2017 and March 2019–February 2022.

Season	Home range size (ha)	SE	Lower CL	Upper CL
Late gestation	875.9	79.4	717.9	1034.0
Calving	627.9	53.5	521.9	734.0
Harems	694.1	45.2	604.4	783.8
Mid-gestation	586.4	47.3	492.2	680.6

### Yearly and seasonal land cover and home range size

We observed collinearity (|r|> 0.5) in land cover types among our seasonal and yearly datasets. For all seasons, the proportions of mines were negatively correlated with both oak forests and mixed hardwood forests. During mid-gestation, late gestation, and calving seasons and year-round, the proportions of oak and mixed hardwood forests were positively correlated. Finally, mixed hardwood forests and NM open land were negatively correlated during mid-gestation season. Additionally, we had no competing models for any seasonal or annual evaluation of home range size relative to land cover composition (Table 2).

**Table 2:** Top mixed-effect models and their corresponding degrees of freedom (df), Akaike information criterion (AIC), and delta AIC (ΔAIC) values examining elk space use as a function of cover type in southwestern VA, from June 2012–Feb 2017 and March 2019–February 2022.

Season	Model	df	AIC	ΔAIC
Late gestation	**Mixed hardwood + NM open land**	**5**	**1139.90**	**0.00**
	NM open land + Mines	5	1166.10	26.20
	Mixed hardwood	4	1166.29	26.39
	Oak + NM open land	5	1187.38	47.48
	NM open land	4	1199.25	59.35
	Mines	4	1220.98	81.08
	Oak	4	1227.75	87.85
	Null	3	1244.69	104.79
Calving	**Mixed hardwood + NM open land**	**5**	**1564.59**	**0.00**
	Oak + NM open land	5	1570.71	6.12
	NM open land + Mines	5	1571.39	6.80
	Mixed hardwood	4	1576.90	12.31
	NM open land	4	1579.90	15.31
	Mines	4	1591.86	27.27
	Oak	4	1593.32	28.73
	Null	3	1601.34	36.75
Harems	**Oak + Mixed hardwood + NM open land**	**6**	**1254.48**	**0.00**
	Mixed hardwood + NM open land	5	1277.29	22.81
	Oak + Mixed hardwood	5	1277.52	23.05
	Oak + NM open land	5	1279.64	25.16
	NM open land + Mines	5	1286.76	32.29
	NM open land	4	1294.00	39.52
	Mixed hardwood	4	1294.02	39.54
	Oak	4	1320.01	65.53
	Mines	4	1320.03	65.55
	Null	3	1327.45	72.98
Mid-gestation	**NM open land + Mines**	**5**	**1137.87**	**0.00**
	Oak + NM open land	5	1141.15	3.28
	NM open land	4	1149.49	11.62
	Mixed hardwood	4	1150.08	12.21
	Oak	4	1166.52	28.66
	Mines	4	1168.47	30.60
	Null	3	1180.67	42.81
Year-round	**Mixed hardwood + NM open land**	**5**	**5210.30**	**0.00**
	Mixed hardwood	4	5220.53	10.22
	NM open land + Mines	5	5249.46	39.16
	Oak + NM open land	5	5269.36	59.05
	NM open land	4	5276.19	65.89
	Mines	4	5285.18	74.87
	Oak	4	5306.85	96.54
	Null	3	5328.28	117.97

Models ranked based on delta AIC with bolded models used as the best models. We used four land cover types in our models: oak forests (oak), mixed hardwood forests (mixed hardwood), land associated with mining (mines) and non-mined open land (NM open land) with the null model including no land cover types.

**Figure 3:**
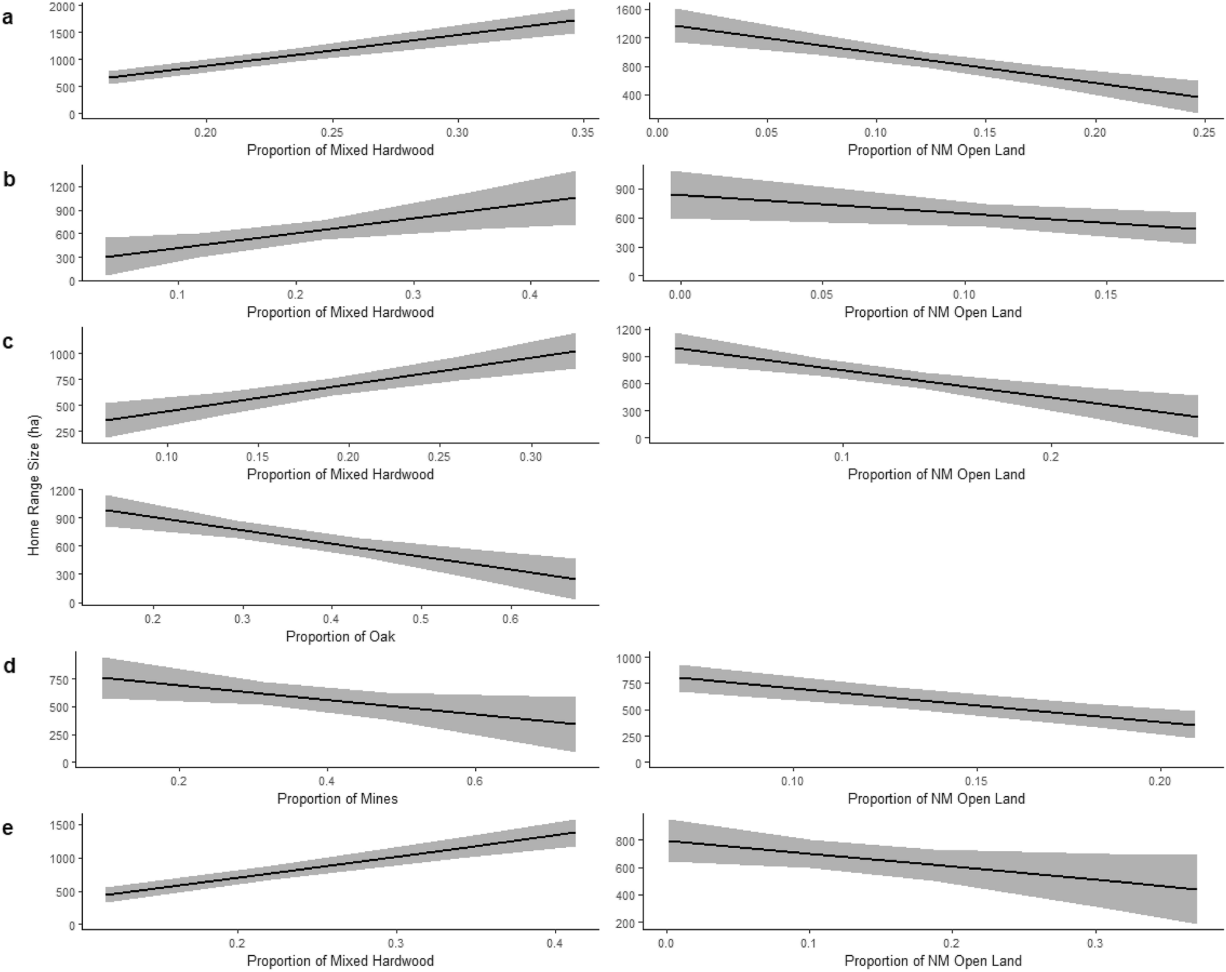
Prediction plots of individual top model parameters explaining the relationship between female elk home range size in hectares (ha) and proportions of land cover within the home range during late gestation (**a**), calving (**b**), harems (**c**), and mid-gestation (**d**) seasons and year-round (**e**) in southwestern Virginia, USA from June 2012–February 2017 and March 2019–February 2022. The top model for each season and year-round included two land cover types as the best predictors of home range size except for harems season. Proportions of mixed hardwood and non-mined (NM) open land were the strongest predictors during late-gestation and calving seasons and year-round. In addition to mixed hardwood and NM open land, proportion of oak was also included in the best model during harems. The top model for mid-gestation included the proportions of mines and NM open land.

Across all elk biological seasons, all open land cover types in the top models had a negative relationship with home range size, and all forested land cover types had a positive relationship except oak during the harems season (Table 3; Fig. 3). Presented effect sizes are given as 5% increases of respective land cover types as 5% was close to one standard deviation for all land cover types. The effect size of increasing the proportion of mixed hardwood forests by 5% varied by season ranging from increasing home range size by 93.9 ha to 284.2 ha during calving and late gestation seasons, respectively (Fig. 3). Notably, the proportion of mixed hardwood forests was not included in the best model for mid-gestation; but instead, included non-forested habitat types (mines and NM open land; Table 3) for which NM open land had a had a larger effect size on home range size than mines—decreasing home range size by 159.6 ha compared to 32.9 ha with a 5% increase in their respective proportions (Fig. 3). Every season and year round’s top models included NM open land (Table 3). For every 5% increase in NM open land home range size decreased by 48.5 ha to 204.7 ha across seasons (Fig. 3). Harems season was the only season to include more than two land cover proportions in its top model (Table 3; Fig. 3). In addition to the proportions of mixed hardwood and NM open land, harems season was the only season to include the proportion of oak forests for which a 5% increase resulted in a 69.5 ha decrease in seasonal home range size (Table 3; Fig. 3).

**Table 3:** Top linear mixed-effects models examining home range size as a function of land cover types in southwestern Virginia, USA from June 2012–February 2017 and March 2019–February 2022 based on delta Akaike information criterion.

Season	Model	Term	Estimate	SE	Pr( >|t|)
Late gestation	Mixed hardwood + NM open land	(Intercept)	872.79	53.63	< 0.001
		Mixed hardwood	421.16	49.27	< 0.001
		NM open land	− 252.43	52.54	< 0.001
Calving	Mixed hardwood + NM open land	(Intercept)	603.50	58.23	< 0.001
		Mixed hardwood	150.39	54.19	0.007
		NM open land	− 117.97	55.68	0.037
Harems	Oak + Mixed hardwood + NM open land	(Intercept)	688.17	41.57	< 0.001
		Oak	− 146.55	35.15	< 0.001
		Mixed hardwood	168.66	37.29	< 0.001
		NM open land	− 152.29	36.29	< 0.001
Mid-gestation	Mines + NM open land	(Intercept)	591.67	48.81	< 0.001
		Mines	− 235.42	44.48	< 0.001
		NM open land	− 84.44	38.02	0.029
Year-round	Mixed hardwood + NM open land	(Intercept)	678.44	50.49	< 0.001
		Mixed hardwood	233.13	28.87	< 0.001
		NM open land	− 59.68	29.90	0.047

We used four land cover types in our models: oak forests (oak), mixed hardwood forests (mixed hardwood), land associated with mining (mines) and non-mined open land (NM open land).

Based on our seasonal and year-round top models, the best predictor of home range size was mixed hardwood, followed by NM open lands, oak forests, and mines (oak forests and mines only included in harems and mid-gestation models, respectively; Table 3; Fig. 3). Although all top models included NM open lands, when modeled individually, mixed hardwood had lower delta AIC than NM open land during late gestation and calving seasons and year-round. When included together, mixed hardwood always had a greater significance and greater effect size on home range size than NM open lands.

## Discussion

We found support for our hypothesis that elk space use would vary as a function of habitat composition and spatiotemporal variation in resources across the annual cycle. Our results supported our first prediction that elk home range size would be inversely related to the proportion of non-forested habitats and positively related to forested habitats. However, we did not find support for our second prediction of elk home range size increasing during the dormant, or mid-gestational, season. Mechanistically, elk likely restricted their movements during less productive times of year (leaf-off dormant season), whereas throughout the year, greater proportions of open habitats and forested habitats decreased and increased their space use, respectively.

Considering the habitat productivity hypothesis, our results suggest open areas are likely higher quality for elk than forested cover because home range sizes decreased with increasing proportions of all open lands. However, the harems season’s top model was the only seasonal model to include oak forests, which had a negative relationship with home range size (Table 3; Fig. 3). During this time of year, acorns are a food resource used by eastern elk. For example, in eastern Kentucky, elk increased their woody browse intake during fall, however they did not differentiate leafy forage from hard mast^36^. However, another study found 10% of fall elk diets in Tennessee were comprised of acorns^35^. Our findings suggest that female elk in southwestern Virginia may also take advantage of this seasonal food resource. Although in support of our prediction, mined areas appeared to have little influence over space use throughout the year (Table 3; Fig. 3). However, this is not to say mines do not impact home range size. Due to collinearity amongst habitat proportions, the proportion of mines could not be included in the same model with the majority of the other habitat types. As a result, the combination of multiple habitat types, i.e., the proportions of mixed hardwood and NM open land were better predictors of home range size than the proportion of mines alone. Yet, mines were included during mid-gestation for which their proportion had a negative relationship with home range size (Table 3; Fig. 3). During mid-gestation, reclaimed mines likely serve as the vital food resource for elk^35,36^.

Despite acorns serving as a seasonal resource, our results suggest that mixed hardwood forests may be poor habitat for female elk throughout the year. Mixed hardwood remained the most significant land cover type in predicting home range size suggesting poor quality habitats may be more influential in predicting space use than high quality ones. This may denote that the avoidance of habitat may influence home range size more than selection in our study area. Alternatively, forests may not be of poor quality per se, just the most abundant in the study area^10^ as forested cover types accounted for 75% of our land area. Thus, as elk increased their home range size, their range invariably included larger proportions of mixed hardwood forests regardless of the mechanism driving increased space use. When food resources are abundant or conspecific density is low, search and movement choices are often not as important because individuals are not energetically limited by resources or interspecific competition^66^. Therefore, it is possible that the density of elk is low relative to the carrying capacity of our study area; thus, animals may be released from density-dependent effects observed in other regions^67^.

Contrary to our prediction that home range sizes would increase during the dormant season, we found the smallest home ranges during mid-gestation, or winter, (Table 1) when forage palatability and availability is lowest for the habitats most prevalent on our landscape^36^. These conflicting results may be explained through the lens of movement economy theory^66^—wherein during periods of resource scarcity, individuals will adjust activity budgets to expend as little energy as possible. During winter, elk are in an nutritional deficit due to low resource abundance, and movements associated with food searching may increase this deficit further depleting fat reserves gained during summer and fall^2^. Indeed, these findings align with work in western systems wherein elk activity and movement decreased during winter presumably due to lack of quality forage^68,69^. In conjunction with movement economy theory and findings elsewhere, our results may reflect optimal elk movement to minimize energy expenditure during a biological period with low resource abundance.

Energetic needs associated with life-history phenology and forage quality may also drive elk activity and movement. Our largest home ranges occurred during late gestation, a period characterized by peak forage quality and quantity in our study area^45^. However, during gestation female elk’s energetic requirements increase^70^ and individuals must consume a greater volume and higher quality forage to meet these requirements to sustain pregnancy. Consequently, females may increase their movements and activity during gestation in search of suitable forage^3,71^ resulting in increased home ranges as observed in roe deer (*Capreolus capreolus*)^8^ and fallow deer (*Dama dama*)^72^. Additionally, female elk often will go on excursions for maternal isolation, in some instances moving > 2.5 km in the days leading to parturition^73^.

In contrast, forage biomass may be more important than forage quality for graminoid specialists during some biological seasons^74^. Following spring green-up, forage becomes more fibrous and less digestible, effectively lowering its quality^75^; yet absolute forage availability increases during summer. After parturition, female’s energetic requirements are at their yearly highest due to the demands of lactation^70^ and may result in greater space use as females search for high quality forage. However, due to their body size, and corresponding ruminant gut capacity, elk can extract more nutrients from poorer quality forages compared to smaller herbivores^74,76,77^. Consequently, this may indicate that during the calving season elk have access to greatest forage biomass and diversity in our study area^35^, which may result in reduced home range sizes as supported by our findings (Table 1) and suggested elsewhere^78^.

We emphasize that our analyses excluded topographic variables (i.e., terrain roughness, slope, aspect, and elevation) and habitat types with low proportions within the home ranges (i.e., conifer forests, developed, and water). Given these variables were excluded, we may have overlooked species-habitat relationships non-sequitur to our hypothesis that have been documented elsewhere. For example, within our study area conifer forests (1.3% of study area) may serve as important cover to buffer temperature extremes (e.g., extreme heat or cold)^12^. Further, female elk have shown to decrease home range size as a function of development (6.7% of study area)^20^. Thus, within our study area, elk may avoid developed areas due to human activity despite having fertilized lawns and horticultural cultivars of high palatability and crude protein^10,14,22^. Finally, in other regions, elk have shown to modulate habitat selection relative to slope, as topography may be an important driver of forage^15^. Importantly, within our study area mixed hardwoods are characterized by very steep and rugged terrain, contrastingly, open cover corresponds to gentler topography, whether naturally or a function of post-mining reclamation^22,79^. As a result, elk may avoid the rugged terrain heavily associated with mixed hardwood forest, not necessarily the habitat type itself.

We caution that our findings are relevant only to the female elk. For sexually dimorphic ungulates, males and females often differ in their space and habitat use and energetic requirements. Generally, males can consume lower quality, but more abundant forage throughout the year due to their larger gut capacity^71,80,81^. However, metabolic demand fluctuates throughout the year for male and female ungulates (i.e., female gestation and lactation, male antler growth, pre-rut hyperphagia, and rut recovery)^71,80,81^. For instance, female elk activity in southeastern Kentucky was considerably higher than male activity during winter and spring in preparation for parturition^82^. Because of differing energy requirements throughout the year, male and female elk may respond differently to habitat productivity. Female white-tailed deer (*Odocoileus virginianus*) do not follow the habitat productivity hypothesis as closely as males wherein female space use is driven by other factors such as fawning^71^. Similarly, male mule deer (*Odocoileus hemionus*) home range sizes decreased as productivity increased, whereas female’s home range sizes had no detectable relationship with productivity^83^. Accordingly, future research in our study area should include females and males across biological seasons.

An animal’s space use and habitat therein may be used as a proxy for habitat quality^3,23^. Thus, linking home range size to land cover promotes a deeper understanding regarding how animals utilize their landscape. A better understanding of space use relative to habitat types is particularly important for species reintroductions as understanding spatiotemporal variation in habitat quality is essential for selecting reintroduction sites, identifying potential range expansion, and mitigating human-wildlife conflicts. We found habitat requirements for a restored elk population varied seasonally following both biological cycles and annual cycles of forage quality and availability, and open habitats were consistently related to smaller home ranges suggesting higher quality than forested ones for elk in our system. We observed the largest seasonal home ranges for female elk during the last two months of gestation, likely the result of pre-partum restlessness and increasing forage consumption due to maternal energetic requirements. Currently, elk are not limited by their habitat requirements in southwestern Virginia. However, this research provides insight into the spatial requirements of elk and identifies habitat types managers should prioritize the acquisition and creation of through private land management programs to promote elk habitat.

## Data Availability

The datasets generated during and/or analyzed during the current study are available from the corresponding author upon reasonable request.
